# *Mycobacterium tuberculosis* infection following polyacrylamide hydrogel (Amazingel) breast augmentation: A case report and literature review

**DOI:** 10.1097/MD.0000000000043947

**Published:** 2025-08-15

**Authors:** Jianfei Zhang, Du Zhang, Kaixi Tan, Bin Jiang

**Affiliations:** a Department of Burns and Plastic Surgery, The Second Affiliated Hospital, Hengyang Medical School, University of South China, Hengyang City, Hunan Province, China; b Department of Burns and Plastic Surgery, The Affiliated Nanhua Hospital, Hengyang Medical School, University of South China, Hengyang City, Hunan Province, China.

**Keywords:** Amazingel, infection, *Mycobacterium tuberculosis*, polyacrylamide hydrogel

## Abstract

**Rationale:**

Polyacrylamide hydrogel (PAAG) injection for breast augmentation is linked to long-term complications like inflammation and infection, with bacterial infections being well-documented. However, mycobacterial infections following such procedures are extremely rare, making this case clinically significant for enhancing awareness of unusual pathogens in post-PAAG complications.

**Patient concerns:**

A 42-year-old female reported persistent right breast pain, and the wound failed to heal for a long time after surgery to remove the injected material, 15 years after undergoing PAAG injection for breast augmentation. These symptoms persisted despite prior interventions, causing significant discomfort and prompting further medical evaluation.

**Diagnoses:**

The initial assessment misdiagnosed the condition as a bacterial infection. However, subsequent acid-fast bacilli staining of wound secretions and TBseq Ultra-gene sequencing confirmed the presence of *Mycobacterium tuberculosis*, leading to a definitive diagnosis of mycobacterial infection.

**Interventions:**

The patient received a 2-month intensive anti-tuberculosis regimen consisting of rifampicin, isoniazid, ethambutol, and pyrazinamide. This targeted therapy was initiated following the confirmation of *M tuberculosis* infection.

**Outcomes:**

After completing the 2-month anti-tuberculosis treatment, the patient achieved complete wound healing. A 3-month follow-up period showed no recurrence of symptoms, indicating successful resolution of the infection.

**Lessons:**

This case emphasizes that *M tuberculosis* should be considered a potential pathogen in refractory soft tissue infections following PAAG injection. Clinicians must maintain a high index of suspicion for mycobacterial infections when wounds fail to respond to standard therapies. Early use of acid-fast staining and molecular diagnostics (e.g., gene sequencing) is critical for timely and accurate diagnosis, enabling targeted treatment to improve patient outcomes.

## 1. Introduction

Polyacrylamide hydrogel (PAAG), marketed as *Amazingel*, has been utilized in cosmetic breast augmentation due to its viscoelastic properties and purported biocompatibility.^[1]^ However, long-term complications (including migration, granuloma formation, and infection) have raised significant safety concerns.^[2]^ While bacterial infections following PAAG injections are well-documented, mycobacterial infections remain exceedingly rare, with fewer than 10 cases reported globally.^[3]^

*Mycobacterium tuberculosis* (MTB), the causative agent of TB, predominantly affects the lungs but may manifest in extrapulmonary sites in 15% to 20% of cases.^[4]^ Cutaneous and soft tissue MTB infections are particularly uncommon, accounting for only 1% to 2% of extrapulmonary TB.^[5]^ These infections often mimic nonspecific inflammatory or bacterial processes, leading to delayed diagnosis and inappropriate therapies.^[6]^ The diagnostic challenge is further compounded by the paucibacillary nature of soft tissue MTB infections, which limits the sensitivity of conventional microbiological methods.^[7]^

Here, we present a case of MTB infection arising 15 years after PAAG breast augmentation. To our knowledge, this represents the first reported instance of MTB complicating PAAG-related soft tissue pathology. This case underscores the importance of considering mycobacterial pathogens in refractory post-procedural infections and highlights the critical role of molecular diagnostics in achieving timely diagnosis.

This study has obtained written informed consent from the patients.

## 2. Case report

### 2.1. Patient history and presentation

A 42-year-old female presented to our department on February 5, 2024, with a 1-year history of progressive right breast pain and non-healing wounds. Fifteen years prior, she had undergone bilateral breast augmentation via injection of polyacrylamide hydrogel (PAAG, Amazingel) at an unlicensed cosmetic clinic. Physical examination revealed multiple irregular, firm masses in the right breast (5–8 cm in diameter), adhering to the skin and underlying musculature, with localized tenderness and mild erythema. No axillary or supraclavicular lymphadenopathy was noted.

### 2.2. Diagnostic workup

During initial evaluation, we comprehensively assessed potential TB sources: the patient denied prior TB exposure, recent travel to endemic regions, or household contact; chest computed tomography (CT) showed no pulmonary involvement.

**Figure 1.**
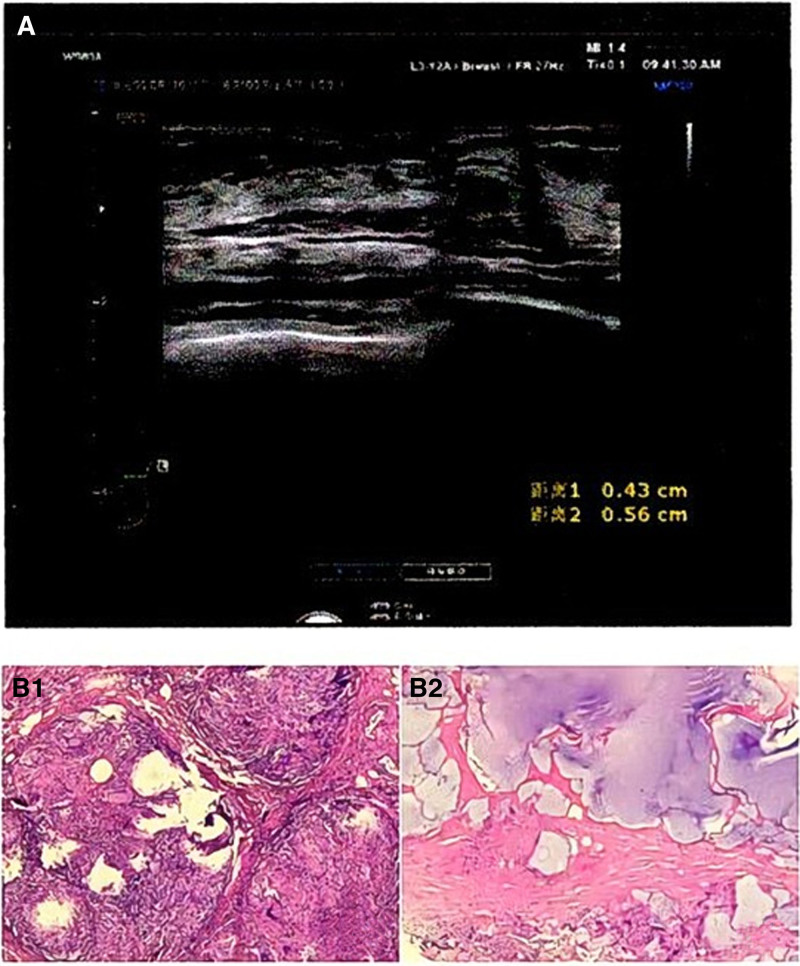
(A) B-ultrasound: (right) multiple cystic dark areas in the breast; (right) multiple hypoechoic areas in the breast and right axilla, considering the possibility of extravasation of breast augmentation fillers. (B) Pathological diagnosis: (right) breast foreign matters and foreign matter granulomas.

Ultrasonography (Fig. 1A) demonstrated multiple cystic lesions (3–5 cm) and hypoechoic areas within the right breast parenchyma and axilla, suggestive of PAAG leakage and granulomatous inflammation. Routine blood tests (WBC: 6.2 × 10⁹/L; C-reactive protein [CRP]: 12 mg/L) showed no signs of systemic infection.

### 2.3. Surgical intervention

On February 6, 2024, surgical excision of the injected material was performed under local anesthesia. Intraoperative findings included extensive yellow granular deposits (Fig. 1B) and fibrotic adhesions. Histopathology confirmed foreign body granulomas with multinucleated giant cells and chronic inflammatory infiltrates. Bacterial cultures of excised tissue and drainage fluid remained negative.

### 2.4. Postoperative course and diagnosis

Despite daily wound irrigation and empiric antibiotic therapy (cefazolin 2 g/day for 14 days), the patient developed worsening inflammation (CRP: 68 mg/L; erythrocyte sedimentation rate: 45 mm/h) with persistent serosanguinous discharge. On April 10, 2024, acid-fast staining of wound secretions revealed rare acid-fast bacilli (1–2/high power field). Subsequent TBseq Ultra sequencing (Illumina platform) identified *M tuberculosis* complex (MTBC) with 1281 unique genomic reads, confirming MTB infection (Table 1). No evidence of pulmonary TB was found on chest CT.

**Table 1:** TBseq Ultra-gene identification results.

Classification	Test result	Sequence numbers
*Mycobacterium tuberculosis* Complex (MTBC)	Positive	1281
Non-tuberculous mycobacteria (NTM)	Negative	–

### 2.5. Treatment and outcome

A standardized 2-month intensive anti-TB regimen was initiated:

Rifampicin: 600 mg/day
Isoniazid: 300 mg/day
Ethambutol: 1200 mg/day
Pyrazinamide: 1500 mg/day

Within 4 weeks, wound exudate decreased significantly, and CRP normalized (5 mg/L). Complete epithelialization was achieved by the end of the second month. At 3-month follow-up, the patient remained asymptomatic with no recurrence.

## 3. Discussion

This case illustrates a rare yet critical complication of PAAG-based breast augmentation: delayed soft tissue infection caused by MTB. To our knowledge, this represents the first reported instance of MTB infection associated with PAAG injection, expanding the spectrum of long-term complications linked to this controversial filler material.

### 3.1. Diagnostic challenges and mechanistic insights

MTB infections of the skin and subcutaneous tissue account for <2% of extrapulmonary TB cases, often mimicking nonspecific bacterial infections or foreign body reactions.^[5]^ In our patient, the initial misdiagnosis as a routine PAAG-induced inflammatory response highlights 2 key diagnostic pitfalls:

1. Latency of presentation: the 15-year interval between PAAG injection and symptom onset contrasts sharply with typical bacterial infections, which usually manifest within months.^[8]^ This prolonged latency may reflect either reactivation of dormant MTB or delayed contamination through hematogenous spread from a latent pulmonary focus, though chest CT revealed no active pulmonary involvement.
2. Paucibacillary nature: the scarcity of acid-fast bacilli (1–2/high power field) in wound secretions underscores the limitations of conventional staining methods. Molecular diagnostics (e.g., TBseq Ultra sequencing) proved indispensable, detecting MTB-specific genomic sequences despite low bacterial burden: a finding consistent with prior studies on paucibacillary TB.^[9]^

### 3.2. Clinical implications

The successful resolution of infection following a 2-month intensive anti-TB regimen aligns with WHO guidelines for extrapulmonary TB,^[10]^ suggesting that soft tissue MTB infections may require shorter treatment durations compared to pulmonary disease. However, this hypothesis warrants validation in larger cohorts. Notably, surgical debridement alone failed to control the infection, emphasizing the necessity of combined surgical and pharmacological interventions in such cases.

### 3.3. PAAG safety revisited

While PAAG was initially marketed as a “biocompatible” filler, accumulating evidence associates it with granuloma formation (78% of cases), migration (32%), and late-onset infections (15%).^[2]^ Our case adds MTB to the list of pathogens capable of exploiting PAAG-induced tissue damage. The hydrogel’s non-degradable nature may create a biofilm-friendly microenvironment,^[11]^ facilitating persistent colonization by slow-growing organisms like MTB: a mechanism previously observed in *Mycobacterium abscessus* infections post-liposuction.^[12]^

Unlike the more common *M abscessus* infections post-PAAG: which typically present within 6 months with biofilm-driven pathology requiring aggressive debridement and macrolide-based regimens: this MTB case is the first reported instance linked to PAAG, exhibiting a uniquely prolonged 15-year latency suggesting reactivation (rather than acute inoculation), absence of documented biofilm dependence, and rapid resolution with standard anti-TB drugs alone. This contrast underscores MTB’s distinct pathogenesis as a late reactivation phenomenon in PAAG complications, warranting vigilance in TB-endemic regions despite its rarity.

### 3.4. Limitations

This study has several limitations:

- The absence of MTB strain genotyping precludes definitive identification of the infection source (exogenous vs reactivation).
- Long-term follow-up (beyond 3 months) is needed to assess recurrence risks.
- Generalizability is limited by the single-case design.

## 4. Conclusion

Clinicians managing refractory soft tissue infections post-PAAG injection should maintain a high index of suspicion for mycobacterial pathogens. Early utilization of acid-fast staining and gene sequencing can avert diagnostic delays, while tailored anti-TB therapy (guided by molecular diagnostics) offers a curative pathway. Regulatory agencies must reassess the safety profile of PAAG in light of its potential to harbor atypical pathogens.

## Author contributions

**Conceptualization:** Jianfei Zhang, Bin Jiang.

**Data curation:** Du Zhang.

**Resources:** Jianfei Zhang, Kaixi Tan.

**Writing – original draft:** Jianfei Zhang, Du Zhang, Kaixi Tan.

**Writing – review & editing:** Bin Jiang.
